# United Kingdom Frozen Shoulder Trial (UK FROST), multi-centre, randomised, 12 month, parallel group, superiority study to compare the clinical and cost-effectiveness of Early Structured Physiotherapy versus manipulation under anaesthesia versus arthroscopic capsular release for patients referred to secondary care with a primary frozen shoulder: study protocol for a randomised controlled trial

**DOI:** 10.1186/s13063-017-2352-2

**Published:** 2017-12-22

**Authors:** Stephen Brealey, Alison L. Armstrong, Andrew Brooksbank, Andrew Jonathan Carr, Charalambos P. Charalambous, Cushla Cooper, Belen Corbacho, Joseph Dias, Iona Donnelly, Lorna Goodchild, Catherine Hewitt, Ada Keding, Lucksy Kottam, Sarah E. Lamb, Catriona McDaid, Matthew Northgraves, Gerry Richardson, Sara Rodgers, Sarwat Shah, Emma Sharp, Sally Spencer, David Torgerson, Francine Toye, Amar Rangan

**Affiliations:** 1 York Trials Unit, Department of Health Sciences, Faculty of Sciences, University of York, ARRC Building, York, YO10 5DD UK; 2 University Hospitals of Leicester NHS Trust, Leicester General Hospital, Gwendolen Road, Leicester, LE5 4PW UK; 3 Glasgow Royal Infirmary, 84 Castle Street, Glasgow, G4 0SF UK; 4 Nuffield Department of Orthopaedics Rheumatology and Musculoskeletal Sciences, University of Oxford, Botnar Research Centre, Windmill Road, Oxford, OX3 7LD UK; 5 Blackpool Victoria Hospital and Blackpool and School of Medicine University of Central Lancashire, Preston, PR1 2HE UK; 6 Nuffield Department of Orthopaedics, University of Oxford, Botnar Research Centre, Headington, Oxford, OX3 7LD UK; 7 University Hospitals of Leicester NHS Trust, Gwendolen Road, Leicester, LE5 4PW UK; 8 University of Glasgow, Glasgow Royal Infirmary Orthopaedic Research Unit, Gatehouse Building, 84 Castle Street, Glasgow, G4 0SF UK; 9 James Cook University Hospital, South Tees Hospitals NHS Trust, Marton Road, Middlesbrough, TS4 3BW UK; 10 Department of Trauma and Orthopaedics, South Tees Institute of Learning Research and Innovation, The James Cook University Hospital, South Tees Hospitals NHS Foundation Trust, Marton Road, Middlesbrough, TS4 3BW UK; 11 Nuffield Department of Orthopaedics, Rheumatology and Musculo-skeletal Sciences, University of Oxford, Botnar Research Centre, Oxford, Ox4 7AL UK; 12 Centre for Health Economics, University of York, York, YO10 5DD UK; 13 Department of Health Sciences, Faculty of Sciences, University of York, Seebohm Rowntree Building, York, YO10 5DD UK; 14 Orthopaedic Department, Glasgow Royal Infirmary, 84 Castle Street, Glasgow, G4 0SF UK; 15 Edge Hill University, St. Helens Road, Ormskirk, Lancashire L39 4QP UK; 16 Nuffield Orthopaedic Centre, Oxford University Hospitals NHS Foundation Trust, Windmill Road, Headington, Oxford, OX3 7HE UK; 17 Department of Health Sciences, University of York, York, YO10 5DD UK; 18 Faculty of Medical Sciences and NDORMS, University of Oxford, Oxford, OX3 7LD UK

**Keywords:** Frozen shoulder, Physiotherapy, Manipulation under anaesthesia, Arthroscopic capsular release, Randomised controlled trial

## Abstract

**Background:**

Frozen shoulder (also known as adhesive capsulitis) occurs when the capsule, or the soft tissue envelope around the ball and socket shoulder joint, becomes scarred and contracted, making the shoulder tight, painful and stiff. It affects around 1 in 12 men and 1 in 10 women of working age. Although this condition can settle with time (typically taking 1 to 3 years), for some people it causes severe symptoms and needs referral to hospital. Our aim is to evaluate the clinical and cost-effectiveness of two invasive and costly surgical interventions that are commonly used in secondary care in the National Health Service (NHS) compared with a non-surgical comparator of Early Structured Physiotherapy.

**Methods:**

We will conduct a randomised controlled trial (RCT) of 500 adult patients with a clinical diagnosis of frozen shoulder, and who have radiographs that exclude other pathology. Early Structured Physiotherapy with an intra-articular steroid injection will be compared with manipulation under anaesthesia with a steroid injection or arthroscopic (keyhole) capsular release followed by manipulation. Both surgical interventions will be followed with a programme of post-procedural physiotherapy. These treatments will be undertaken in NHS hospitals across the United Kingdom. The primary outcome and endpoint will be the Oxford Shoulder Score (a patient self-reported assessment of shoulder function) at 12 months. This will also be measured at baseline, 3 and 6 months after randomisation; and on the day that treatment starts and 6 months later. Secondary outcomes include the Disabilities of Arm Shoulder and Hand (*Quick*DASH) score, the EQ-5D-5 L score, pain, extent of recovery and complications. We will explore the acceptability of the different treatments to patients and health care professionals using qualitative methods.

**Discussion:**

The three treatments being compared are the most frequently used in secondary care in the NHS, but there is uncertainty about which one works best and at what cost. UK FROST is a rigorously designed and adequately powered study to inform clinical decisions for the treatment of this common condition in adults.

**Trial registration:**

International Standard Randomised Controlled Trial Register, ID: ISRCTN48804508. Registered on 25 July 2014.

**Electronic supplementary material:**

The online version of this article (doi:10.1186/s13063-017-2352-2) contains supplementary material, which is available to authorized users.

## Background

A large, United Kingdom (UK)-based primary care study found that ‘frozen shoulder’ affects 8.2% of men and 10.1% of women of working age [1]. A shoulder surgeon’s hospital care experience in the UK, however, suggests that the term frozen shoulder is often overused and misused, with incidence in the general population around 1% [2]. Although viewed as a self-limiting condition, long-term follow-up data are scarce [3]. Based on a series of 233 patients with a mean follow-up of 4.4 years from onset of symptoms, 59% had normal or near normal shoulders, 35% had mild-to-moderate symptoms with pain being the most common complaint and 6% had severe symptoms at follow-up [4]. Recent systematic reviews have identified large gaps in the evidence-base and uncertainty in the effectiveness of treatments for frozen shoulder and a need for high-quality primary research [5, 6]. From searching the Health Technology Assessment (HTA) website and the ISRCTN register, there was no large-scale, multi-centre, randomised controlled trial (RCT) of interventions for primary frozen shoulder being undertaken.

The aim of our research is to provide evidence of clinical and cost-effectiveness for commonly used interventions in the National Health Service (NHS) for the management of frozen shoulder in secondary care. We used the findings of a national survey of health care professionals [7] to inform the decision to compare Early Structured Physiotherapy (ESP) and intra-articular steroid injection with the two most frequently used and more costly surgical interventions, i.e. manipulation under anaesthesia (MUA) and arthroscopic capsular release (ACR). As evidence about patient experiences of a frozen shoulder is limited [5], participants will be interviewed to explore their experience and acceptability of treatment 12 months after enrolment into the study [8]. We will also interview health care professionals about the acceptability of the trial treatments. The objectives are listed in Table 1. The Standard Protocol Items Recommendations for Interventional Trials (SPIRIT) Statement 2013 have been followed for the completion of the protocol (see also Additional file 1: SPIRIT 2013 Checklist: recommended items to address in a clinical trial protocol and related documents).

**Table 1:** UK FROST trial objectives

Objectives	
1	The primary objective is to determine the effectiveness of ESP versus MUA versus ACR for patients referred to secondary care for treatment of primary frozen shoulder. This will be achieved using as a parallel-group RCT and, as our primary outcome, the Oxford Shoulder Score (OSS) which is a patient-reported outcome measure at 3, 6 and 12 months. The primary time point is 12 months after randomisation
2	To compare the cost-effectiveness of the three management policies, to identify the most efficient provision of future care, and to describe the resource impact that various policies for frozen shoulder management will have on the NHS
3	To qualitatively explore the acceptability of the different treatments to patients and health care professionals and to provide important patient-centred insight to further guide clinical decision-making
4	To update the HTA-funded systematic review of management of the frozen shoulder for RCT evidence of the effectiveness of these interventions in secondary care. This will allow our findings to be considered in the context of existing evidence on all treatments of interest for this condition
5	To use networks of health care professionals, patients, health service managers and commissioning groups to widely disseminate the findings of this study. This will be in addition to publishing the results of the study in key journals and publishing the HTA report

*ACR* arthroscopic capsular release*, ESP* Early Structured Physiotherapy, *HTA* Health Research Authority, *MU*A manipulation under anaesthesia

## Methods

### Trial design

UK FROST is a randomised, controlled, multi-centre superiority trial comparing three parallel groups (ESP versus MUA versus ACR) for patients referred to secondary care for treatment of primary frozen shoulder. The primary outcome and endpoint will be the Oxford Shoulder Score at 12 months after enrolment into the study. Computer-generated randomisation will be performed using permuted blocks of random sizes, stratified by the presence of diabetes, with unequal random allocation (1:2:2; ESP:MUA:ACR). To reduce the risk of allocation prediction we will not stratify by centre. We will include a concomitant economic evaluation and a nested qualitative study with trial participants and health care professionals. An internal pilot study will confirm feasibility.

### Study setting

We estimated that we will need to recruit from 25 NHS hospitals in the UK across a range of urban and rural areas. The pragmatic design of the trial and wide clinician involvement will ensure the applicability and generalisability of study findings. Table 2 lists the hospital sites that will be set up to recruit patients into the trial.

**Table 2:** UK FROST trial participating sites

Study sites	
1	Aberdeen Royal Infirmary
2	Basildon and Thurrock University Hospitals NHS Foundation Trust
3	Bedford Hospital NHS Trust
4	Blackpool Teaching Hospitals NHS Foundation Trust
5	Cardiff and Vale University Health Board
6	Dorset County Hospital NHS Foundation Trust
7	Dudley Group NHS Foundation Trust
8	East and North Hertfordshire NHS Trust
9	East Kent Hospitals University NHS Foundation Trust
10	Forth Valley Royal Hospital
11	Frimley Health NHS Foundation Trust
12	Glasgow Royal Infirmary
13	Hampshire Hospitals NHS Foundation Trust
14	Maidstone and Tunbridge Wells NHS Trust
15	North Bristol NHS Trust
16	North Tees and Hartlepool NHS Foundation Trust
17	Northern Devon Healthcare NHS Trust
18	Northumbria Healthcare NHS Foundation Trust
19	Oxford University Hospitals NHS Trust
20	Perth Royal Infirmary
21	Robert Jones and Agnes Hunt Orthopaedic Hospital NHS Foundation Trust
22	Royal Alexandra Hospital
23	Royal Free London NHS Foundation Trust
24	Royal Liverpool and Broadgreen University Hospitals NHS Trust
25	Sandwell and West Birmingham Hospitals NHS Trust
26	Sherwood Forest NHS Foundation Trust
27	South Tees Hospitals NHS Foundation Trust
28	Southport and Ormskirk NHS trust
29	Taunton and Somerset NHS Foundation Trust
30	The James Paget University Hospital NHS Foundation Trust
31	The Mid Yorkshire Hospitals NHS Trust
32	Torbay and South Devon NHS Foundation Trust
33	United Lincolnshire Hospitals NHS Trust
34	University Hospital Coventry and Warwickshire NHS Trust
35	University Hospital of South Manchester NHS Foundation Trust
36	University Hospitals of Leicester NHS Trust
37	University Hospitals of North Midlands NHS Trust
38	West Glasgow Ambulatory Care Hospital

### Eligibility criteria

Patients with primary frozen shoulder will be identified through clinical examination and plain radiographs [9]. The clinical examination will include the key diagnostic assessment of restriction of passive external rotation in the affected shoulder [10]. There is evidence of good inter-rater agreement on whether restriction is present [11] and a high threshold (50% restriction) for inclusion should sufficiently minimise diagnostic uncertainty. Plain radiographs (antero-posterior and axillary projections) of the affected shoulder will be obtained routinely for all patients to exclude glenohumeral arthritis and other pathology that could lead to similar clinical presentation (e.g. locked posterior dislocation). Table 3 presents the eligibility criteria for participants.

**Table 3:** Patient eligibility criteria

Patients, including diabetics, are eligible for inclusion if they:
1. Are aged 18 years or older
2. Present with a clinical diagnosis of frozen shoulder characterised by restriction of passive external rotation in the affected shoulder to less than 50% of the contralateral shoulder
3. Have radiographs that exclude glenohumeral arthritis and other pathology
Patients will be excluded from this study if:
1. They have a bilateral concurrent frozen shoulder
2. They have a frozen shoulder secondary to trauma, i.e. trauma to the shoulder that required hospital care, e.g. fracture, dislocation, rotator cuff tear
3. They have a frozen shoulder secondary to other causes, e.g. recent breast surgery, radiotherapy
4. Any of the trial treatments are contraindicated, e.g. unfit for anaesthesia or corticosteroid injection
5. They are not resident in a catchment area of a trial site
6. They lack mental capacity to understand the trial or instructions for treatment

The trial team will assess potential sites against criteria (e.g. willingness to allocate treatment based on randomisation, provision of all three treatments, timeliness of delivering surgery etc.) for feasibility to deliver the trial. A qualified physiotherapist (i.e. not a student or assistant) will deliver the physiotherapy. The participating surgeons will be familiar with the surgical procedure(s). There will be no requirements for the minimum number of these surgical procedures that the surgeon needs to perform and no grade of surgeon will be excluded. The participating site will decide who can operate on patients and whether the individual needs to be supervised by a consultant. We will record the level of experience of physiotherapists and surgeons who deliver the trial treatments in terms of their grade and typical number of frozen shoulder patients that they treat.

### Interventions

The components and standardisation of the surgical trial interventions were informed by a survey of 53 surgeons who were principal investigators (PIs) for two multi-centre shoulder surgery RCTs, i.e. PROFHER [12] and UKUFF [13]. Notably, 28 of 35 (80%) responded that they routinely use a steroid injection with MUA; 14 of 46 (30%) routinely provide steroid injection with ACR; 34 of 46 (74%) routinely perform MUA with ACR; and 13 of 46 (28%) and 8 of 46 (17%) surgeons, respectively, routinely release the posterior capsule or perform a subacromial decompression during ACR. The stand-alone physiotherapy (ESP) and the post-procedural physiotherapy programmes were developed using evidence from a systematic review [5], UK guidelines [14], previous surveys of UK physiotherapists [15, 16] and consensus from expert shoulder physiotherapists in secondary care using Delphi methodology [17]. Further details of the physiotherapy programmes will be published separately.

#### Manipulation under anaesthesia with an intra-articular steroid injection

Participants will be placed on the surgical waiting list with routine pre-operative screening. In keeping with NHS waiting list targets, the procedure will be performed within 18 weeks of randomisation under a general anaesthetic usually as a day case. The affected shoulder is manipulated to stretch and tear the tight capsule and to improve range of movement. Surgeons will use an intra-articular injection of corticosteroid to the glenohumeral joint whilst the patient is under the same anaesthetic unless it is contraindicated. Post-operative analgesia, including nerve blocks, will be provided as per usual care in the treating hospital. If the MUA is incomplete, the surgeon will not cross over intra-operatively to capsular release. The details of the procedure will be collected prospectively using a Case Report Form (CRF).

#### Arthroscopic capsular release with MUA

Participants will be placed on the surgical waiting list with routine pre-operative screening for this procedure, which will be performed within 18 weeks of randomisation under a general anaesthetic, usually as a day case. Arthroscopic release of the contracted rotator interval and anterior capsule will be performed, followed by MUA to complete the release of the inferior capsule. Surgeons will use at their discretion additional procedures like posterior capsular release and subacromial decompression. Supplementary steroid injections, which slightly increase the risk of infection and morbidity, will also be used at the surgeon’s discretion [18]. Post-operative analgesia, including nerve blocks, will be provided as per usual care. The details of the procedure will be collected prospectively using a CRF.

#### Nested shoulder capsular tissue and blood samples study

At selected hospitals we will undertake an exploratory nested capsular tissue and blood samples study with the following objectives:

1. To determine molecular processes and cellular abnormalities in tissue obtained during surgery
2. To determine serum protein and cytokine signatures
3. To correlate any tissue and serum abnormalities detected with clinical presentation and response to treatment

The nested study will include patients allocated to ACR with MUA who have not received a steroid injection in the 6 weeks prior to their surgery. When the date for surgery is known, the research nurse (RN) will post a letter to the patient about the nested study, an information leaflet and a consent form. The RN will seek to record written informed consent when the patient attends for their pre-surgery assessment. This is an exploratory study, with no formal power calculation, and plans to include 20 patients.

A tissue sample of capsule from the rotator interval, which is routinely incised or removed as part of ACR, will be obtained for analysis. A venous blood sample will also be collected during surgery. The samples will be fresh-frozen, stored on dry ice and transported securely by courier to the University of Oxford Musculoskeletal BioBank, housed at the Botnar Research Centre, where formal analysis of the capsular tissue will take place.

#### Early Structured Physiotherapy with an intra-articular steroid injection (comparator treatment)

Participants will receive up to 12 sessions of structured physiotherapy over 12 weeks. This will comprise essential ‘focussed physiotherapy’ and optional ‘supplementary physiotherapy’.

The ‘focussed physiotherapy’ package will include an information leaflet containing education, advice on pain management and function; an intra-articular steroid injection and ‘hands-on’ mobilisation techniques—increasingly stretching into the stiff part of the range of movement as the condition improves—for which there is reasonable evidence of effectiveness [19, 20]; and instruction on a graduated home exercise programme progressing from gentle pendular exercises to firm stretching exercises according to stage, which is accepted good practice. All participants randomised to ESP will undergo all elements of this focussed physiotherapy package unless there is a specific clinical reason for them not to do so (e.g. steroid injection in a patient with uncontrolled diabetes; or in a patient with a stiff, but painless, non-irritable shoulder).

Supplementary physiotherapy will comprise those interventions that are not essential, but which are permissible additions, to allow physiotherapists some flexibility. These interventions, which may have been omitted from the national guidelines because they were outside their scope (e.g. acupuncture), and/or because there was a lack of primary academic literature (e.g. hydrotherapy, soft-tissue release techniques), were explored using a Delphi process.

Patients who do not improve with ESP will be referred for further treatment in consultation with the treating clinician at a 12-week assessment. When further treatment after ESP involves surgical intervention, patients will be placed on the normal surgical waiting list. Any further treatment provided will be recorded. We will reimburse the travel expenses for trial participants allocated to ESP. A CRF will be used to record the ESP given at each session (e.g. injection, advice and education, gentle active exercise).

#### Post-procedural physiotherapy (PPP)

Following MUA or ACR, patients will undergo a programme of physiotherapy of up to 12 weeks, normally commencing within 24 h, with the aim of reducing pain and regaining/maintaining the mobility achieved at operation. This PPP is not intended to be identical to ESP because it is applied in a very different context. The research literature is uninformative, but we pre-specified two ‘focussed physiotherapy’ interventions on the basis of established good practice. These are provision of an information leaflet containing education, advice on pain management and function; and instruction on a graduated home exercise programme. All participants randomised to MUA or ACR will undergo all elements of this focussed physiotherapy package unless there is a specific clinical reason for them not to do so. The interpretation of the Delphi survey results was as for ESP. A steroid injection will be avoided during PPP. A CRF will be used to record the PPP given at each session.

#### Steroid injections

The steroid injections will be administered with or without imaging guidance depending on the usual practice of the hospital site as current evidence does not support the superiority of either approach [21].

#### Modifications to interventions

There will be no explicit criteria for modification or discontinuation of the assigned trial treatment. The clinician and participant will discuss whether or not to continue with a treatment for reasons such as poorly controlled diabetes or the treatment no longer being required.

#### Adherence to interventions

Adherence to the trial treatments will be explained in the Trial Site Manual and Site Initiation Visits (SIVs). A requirement of the internal pilot will be to check the feasibility of delivering the ESP programme. This will be extended to include the surgical interventions and PPP. Every month a designated trial coordinator will extract data from the hospital CRFs and update a spreadsheet to record information about aspects of the treatments. The chief investigator (CI), who is a consultant orthopaedic surgeon, and the lead physiotherapist, will review the spreadsheet for treatment adherence and decide whether any action should be taken with a site. This will be further monitored by the Trial Management Group (TMG), the independent Trial Steering Committee (TSC) and the Data Monitoring Ethics Committee (DMEC).

#### Concomitant care

Management of a patient waiting for surgery may include analgesia to ensure pain relief, general advice on care of the arm (e.g. axillary hygiene) and general advice to prevent further stiffness in the limb. This will not include a specific home exercise programme (like that provided with the structured physiotherapy intervention); and a steroid injection will be avoided, as these are considered active interventions.

### Outcomes

#### Primary outcome (Oxford Shoulder Score)

Our primary outcome will be the Oxford Shoulder Score (OSS), a patient-reported measure of functional limitation following shoulder surgery. Development and validation included patients with frozen shoulder [22] and it has been used in the long-term follow-up of these patients [4]. The OSS is a 12-item measure with five response categories and a range of scores from 0 (worst) to 48 (best) [23]. It has been validated against the professionally endorsed Constant Score [24] and the 36-item Short Form Health Survey (SF-36) and responsiveness over a 6-month period following surgical intervention has been established [25]. The OSS will be completed at the hospital at baseline (i.e. day of randomisation) and posted to trial participants at 3, 6 and 12 months after randomisation. The primary endpoint is 12 months after randomisation. The OSS will also be collected at the hospital on the day that treatment starts (i.e. day of the operation or for patients allocated to ESP on the day when the steroid injection is given or first visit to physiotherapy, whichever is the first to be delivered) and posted to participants to complete 6 months from when treatment starts. The OSS is being collected on the day the treatment starts and 6 months later due to the variation in waiting times as to when the trial interventions start.

#### Secondary outcomes

Secondary outcomes will be measured at baseline, 3, 6 and 12 months from randomisation unless otherwise stated.

##### Quick Disabilities of Arm Shoulder and Hand (QuickDASH)

Comparative validity of functional limitation measures is currently unclear for frozen shoulder. We will, therefore, include a well-validated, condition-specific measure for comparison with the OSS. The DASH (Disabilities of the Arm, Shoulder and Hand) is one of the most widely used, well-validated and reliable measures of symptoms and functional limitation in the upper extremity [26]. To minimise responder burden we will use the validated short version, the *Quick*DASH [27]. This 11-item version is scored from 0 to 100 and endorsed by the American Association of Orthopaedic Surgeons [27]. An 8-unit improvement in scores has been defined as the minimum clinically important difference for patients with shoulder problems [28]. Validity and responsiveness for frozen shoulder has been established [29].

##### EuroQol 5 Dimensions (EQ-5D-5 L)

The EQ-5D is a validated, generic and health economic, self-completed, patient-reported outcome measure covering five health domains with three response options [30, 31]. The 5 L version consists of the same five domains as the original EQ-5D-3 L (mobility, self-care, usual activities, pain/discomfort and anxiety/depression), but with five levels rather than three. This is to help overcome problems with ceiling effects and to improve sensitivity [32, 33]. The EQ-5D-3 L has been validated for a range of shoulder conditions [34, 35]. The 5 L version will provide a simple descriptive profile of health status that can be used to estimate quality-adjusted-life-year (QALY) scores in economic evaluations.

##### Pain

Shoulder pain ‘during the past 24 h’ will be measured using the Numeric Rating Scale for pain [36], a single 11-point numeric scale with 0 representing ‘no pain’ and 10 representing ‘worst possible pain’, considered the most valid measure for this population [37].

##### Extent of recovery

To inform the extent of resolution of symptoms over time we will use a simple subjective global question to assess the impact of participants’ frozen shoulder symptoms in the past 24 h on their need for treatment. Responses will be measured using a Visual Analogue Scale from 0 to 100 with best-case (no need to ask for treatment) and worst-case (definitely ask for treatment) anchors.

##### Complications

All complications will be recorded at 12 months for the past year. Infection will be defined as for the ‘Surgical Site Infection’ audit [38]. Delayed wound healing will be defined as any wound that has not healed by 2 weeks. Complex regional pain syndrome will be defined after surgery as pain, swelling and stiffness of the shoulder that has been operated on, and arm and/or hand restricting full tuck of the fingers. Additional complications like nerve, blood vessel, tendon or bone injury; complications related to steroid injection, including steroid flare and septic arthritis, will be recorded.

### Participant timeline

**Fig. 1:**
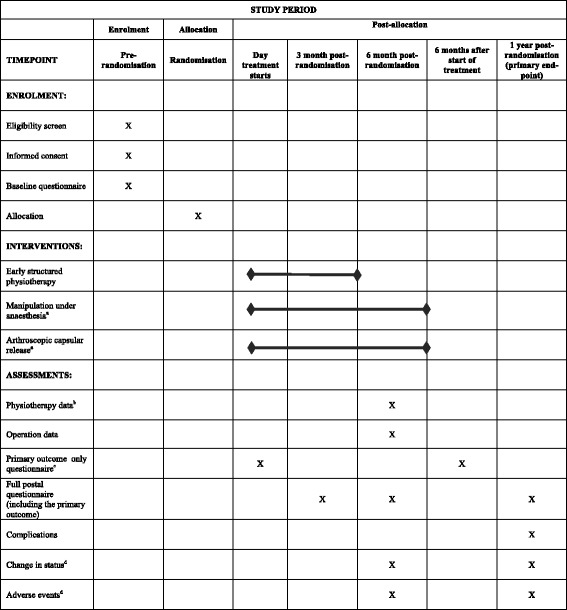
Schedule of enrolment, interventions and assessments for the UK FROST trial. ^a^Patients expected to receive allocated surgical procedure within 18 weeks of randomisation followed by post-procedural physiotherapy. ^b^Physiotherapy logbooks completed recording delivery of Early Structured Physiotherapy (ESP) and post-procedural physiotherapy. ^c^The primary outcome only will be collected on the day treatment starts, i.e. on the day of the patient’s operation or, for patients allocated to ESP, on the day when the steroid injection is given or their first visit to physiotherapy, whichever is the first to be delivered. It will then be collected again 6 months later. ^d^Reminders sent to sites upon return of physiotherapy logbooks and at 1 year to confirm whether any (further) changes in patient status or adverse events need reporting

Figure 1, based on the Standard Protocol Items: Recommendations for Interventional Trials (SPIRIT) Figure, illustrates the overall schedule and time commitment for trial participants from initial eligibility screening, time periods during which trial treatments will be delivered and the data collection/assessments to be performed.

### Sample size

The primary outcome is the OSS. This will be assessed for three treatment comparisons: ESP compared with MUA; ESP compared with ACR (where for both of these comparisons we are testing for a 5-point mean difference on the OSS); and MUA versus ACR (where we are testing for a 4-point mean difference). A 5-point improvement, with a standard deviation (SD) of 12, can be found on the OSS (standard effect size of 0.42) in shoulder patients treated conservatively [39] and with complication-free surgery (author AR, unpublished data, 2014). The developers of the OSS agree that this improvement represents a minimal clinically important difference [23]. This larger effect size will be required to justify the greater costs and potential risks associated with surgery. A smaller mean difference of 4 points on the OSS (standard effect size of 0.33) is expected to distinguish between MUA and ACR. To observe the above effect sizes with 90% power and 5% two-sided significance, adjusting for a conservative estimate (*r* = 0.4) of the correlation between OSS over 12 months and allowing for 20% attrition, a total sample size of 500 patients is required (ESP: 100; MUA: 200; ACR: 200). Owing to the a priori specified sequence of treatment comparisons, multiplicity should not be a concern [40]. Thus, no adjustments are made to the calculation.

### Recruitment

For NHS hospitals in England in 2009/2010 and 2010/2011, using Hospital Episode Statistics that exclude post-trauma or secondary referral from other specialties, there is a stable rate of 210 per million patients treated for frozen shoulder. Assuming that 50% of frozen shoulder patients presenting in secondary care meet the inclusion criteria and 40% consent (based on the PROFHER trial experience comparing surgical versus conservative care in shoulder fracture patients) that leaves around 40 patients per million to be recruited into the trial. To recruit 500 trial participants from trusts that serve around a half-million catchment area we expect to need 25 hospitals to recruit for a minimum of 1 year. This estimate, however, requires no delays in set up or problems at any time after that, all surgeons at a site to participate, and all potential participants to be screened for eligibility. Our experience is that it will be feasible to set up 25 hospitals; however, up to 30 months will be required to meet our recruitment target.

To assess their feasibility to successfully recruit we will ask sites to complete an Expression of Interest Form. The study will be endorsed by the British Elbow and Shoulder Society (BESS) and publicised at the annual BESS conferences. The CI will approach PIs of previous surgical trials of the shoulder (UKUFF and PROFHER) to identify collaborating surgeons and the trial team will also approach PIs at BESS conferences.

Two patients who had been treated for a frozen shoulder at the lead site (James Cook University Hospital) will comment on the patient information leaflet and the consent process for trial participation. The advice of an independent patient representative member of the TSC will also be sought. Following a qualitative study of patients with frozen shoulder using semi-structured interviews [41], we identified the need to develop a leaflet to provide general information about what is a frozen shoulder.

Hospital staff, including an RN normally from the National Institute for Health Research (NIHR) Clinical Research Network, will be provided with training in recruitment at the SIVs. A Trial Site Manual will be prepared for hospital staff which will include guidance on consenting patients into the trial and how to answer questions that might arise during consent. In addition, a poster will be provided to publicise the trial to hospital staff and patients. During the trial, training and reminders will be implemented using regular email bulletins and face-to-face meetings with PIs and RNs. Trial coordinators will provide support and guidance to staff when required.

### Assignment of interventions

The RN or assessing clinician will identify patients who have been referred for a frozen shoulder to an outpatient hospital clinic. In the clinic, a designated individual within the shoulder team (e.g. surgeon, physiotherapist) will complete a CRF to confirm whether the patient is eligible or not; and when applicable, approach the patient about the study. The RN will then provide an information sheet and answer any questions. The patient can agree to consent at that time or take up to a week to decide.

When the patient does not consent, a further CRF will be completed by the RN to briefly record the reason for the patient not consenting and their treatment plan. The patient will also be offered an optional CRF to complete if they would like to provide more information about why they did not take part.

When patients consent and complete their baseline forms, the recruiting clinician will contact York Trials Unit (YTU), either by telephone or via the Internet, to access a secure, computer-generated randomisation service. This will ensure treatment concealment and unbiased allocation. Unequal random allocation (1:2:2 for ESP:MUA:ACR) will be used to allow for the potential difference in effect between treatment comparisons, and stratified by presence of diabetes which is significantly associated with impaired shoulder motion in this patient population [8]. The patient will be informed by the clinician of their treatment allocation.

To ensure concealment we will not stratify by centre and use permuted blocks of random sizes. The randomisation service will record information and check patient eligibility to avoid inappropriate entry of patients into the trial. Patients and the hospital staff will be informed of the allocation. The study office in York will send an allocation letter to the patient explaining what will happen next. The participant’s general practitioner will also receive a letter about treatment allocation. As the trial is pragmatic in design, comparing surgical and non-surgical treatment options, the blinding of participants and clinicians to treatment allocation is not possible.

### Data collection methods

Postal questionnaires will be used to collect data completed by trial participants as already described. In addition, paper CRFs will be used to record all the information required from the protocol that will be collected from the hospital. Each trial participant will have a unique four-digit identification number that will be pre-recorded on all CRFs. There will be an instructions page at the start of each postal questionnaire and for the more complex hospital CRFs. At the SIV we will provide advice on completion of the CRFs including a Trial Site Manual for hospital staff. Active and systematic follow-up of all randomised participants by post will include pre-notification reminders, 2- and 4-week letter reminders and the option to complete an abridged questionnaire (a minimum of the OSS and EQ-5D) via telephone after 6 weeks. At 12 months, the primary time point, we will include an unconditional incentive of £5 [42]. Text messages will be sent on the day that the participant is sent the postal questionnaire [43] and newsletters will be circulated to trial participants [44]. A central database at YTU will manage data collection. A trial participant can entirely withdraw from the study at any time for any reason but any data collected up to that point will be included in the analysis. The participant can also agree to being withdrawn from only postal questionnaire collection or only hospital CRF collection.

### Data management

The patient questionnaires and hospital CRFs will be designed using TeleForm software (version 10; Cardiff Software, Cambridge, UK) and marked up with variable names and appropriate scoring. To maximise data quality, when hospital CRFs are returned to YTU, key variables required for the statistical analysis, and checking adherence in the delivery of the treatments will be reviewed for completion and accuracy by a research data administrator, who will resolve any queries with the RN at the site. The hospital site will be reimbursed for the completion of CRFs which will be signed off by the trust and trial sponsor using a Clinical Trial Agreement. No checks regarding data quality of the postal questionnaires will be made on immediate return to YTU. A trial coordinator will, however, as a duty of care, check whether the participant has responded to the last EQ-5D-5 L question that ‘I am extremely anxious and depressed’ and check free-text responses to questions on whether the participant could be at risk of harm. When this occurs, the PI, RN and CI will be notified by email. After this initial check, all postal questionnaires and hospital CRFs will pass through a process of scanning in the Teleform software, second checking and validation against predetermined rules.

Essential trial documentation will be kept with the Trial Master File and Investigator Site Files. The sponsor will ensure that this documentation is retained for a minimum of 5 years after the conclusion of the trial. The postal questionnaires and hospital CRFs will be stored for a minimum of 5 years after the conclusion of the trial as paper records; and a minimum of 20 years in electronic format [45].

### Statistical methods

The flow of participants through each stage of the trial will be presented in a Consolidated Standards of Reporting Trials (CONSORT) diagram [46]. Unadjusted OSS will be summarised descriptively (*n*, mean, SD, median, minimum and maximum) at each time point by treatment group and overall. To inform treatment selection we will determine (1) whether the two surgical interventions are significantly superior to ESP, and if so, (2) whether key-hole surgery is superior to MUA. Three comparisons will be carried out: ACR versus ESP, MUA versus ESP and ACR versus MUA. All analyses will be carried out using two-sided significance tests at the 0.05 significance level.

Our primary analysis will compare the treatment groups at 12 months. For each of the three treatment comparisons, the primary outcome OSS will be analysed using a linear mixed model, including assessments at all available time points with reference to the date of randomisation (3, 6 and 12 months, thereby increasing power) and treating patients as a random effect. The model will adjust for OSS at baseline and include as fixed effects: treatment arm, time, arm by time interaction and covariates for age, gender and whether diabetic. The model will provide treatment-group differences over 12 months as well as estimates at individual time points. These will be presented as mean estimates with 95% confidence intervals and associated *p* values.. For the modelling of repeated measurements, the best fitting (based on AIC and BIC information criteria), simple (not significantly different from an unstructured pattern) covariance pattern will be selected. Any missing data will be assumed to be missing at random. Model assumptions will be checked and, if they are in doubt, the data will be transformed prior to analysis or alternative non-parametric analysis methods will be used.

The primary analysis will be conducted as intention-to-treat (ITT), including patients in the groups to which they were randomised. To take account of the effects of an expected degree of cross-over, secondary analyses will be carried out using Complier Average Causal Effect analysis at the 12-month time point which retains the initial randomised assignments, thus overcoming the problems of per-protocol analysis [47]. A separate secondary ITT model will include the baseline OSS, OSS on the day treatment starts and OSS 6 months later with the same covariates as the primary analysis to inform the influence of variable treatment waiting times on the results of the study.

Two separate exploratory sub-group analyses will be undertaken: differences in treatment response according to whether the patient has diabetes (yes or no) and whether the patient had received physiotherapy for their affected shoulder prior to enrolment into the trial (yes or no). Simple descriptives of the primary outcome will be reported for the sub-groups. A treatment group by sub-group interaction term will be included in the primary analysis model for each sub-group analysis. For each sub-group analysis, the estimated treatment by sub-group means with associated confidence intervals will be reported along with the *p* value for the interaction term.

To explore the potential effect of patients’ knowledge of which treatment they received (allocation cannot be blinded) and their experience of this treatment on the results of the trial as measured by the primary outcome, we will take two approaches. First, eligible patients will be asked at baseline if they have any treatment preference (physiotherapy, no preference or surgery; and if surgery, which type) and their expectations of the effectiveness of each treatment. These preferences and expectations will be descriptively explored by trial arm as well as for compliant and crossover patients. Separate secondary analyses of the ITT primary outcome model will be conducted including an interaction of the randomised treatment with: treatment preferences, preference rating of the allocated treatment, effectiveness expectations of each treatment and effectiveness rating of the allocated treatment. In addition, at the end of the 12-month follow-up period, patients will again be asked to indicate their treatment preference in the event of a similar shoulder problem given their experience over the past 12 months. Patient preferences at 12 months’ follow-up will be tabulated against baseline preferences and against the allocated treatment. Second, it is possible that patients’ knowledge and experience of treatment may result in non-response at follow-up. A logistic regression model will be used to identify predictors of non-response and will include all baseline data and primary outcome assessments before any missing values. If any variables are found to be predictive of non-response they will be included in the model specified for the primary analysis.

All unadjusted secondary outcomes will be reported descriptively (mean, SD, median, minimum and maximum for continuous data and counts and percentages for categorical data). The following outcomes will be analysed using the same ITT methods as the primary analysis adjusting for the same covariates: *Quick*DASH, pain question and extent of recovery (assessed by a ‘need for further treatment’ rating scale). Separate logistic regression models will be used to determine treatment-group differences in having experienced at least one complication or adverse event.

### Cost-effectiveness analysis

The economic evaluation will determine the relative cost-effectiveness of three interventions for the treatment of frozen shoulder. A cost-utility analysis will compare the incremental health outcome, measured in terms of QALYs, with the incremental cost among the three treatment options. Costs and QALYs will be evaluated from the perspective of the NHS and Personal Social Services, consistent with that used by the National Institute for Health and Clinical Excellence [48].

Health-related quality of life will be assessed during the trial period using the EQ-5D-5 L instrument. The EQ-5D profiles generated for each patient will be valued using a set of estimated preferences based on the UK population that will be generated by the EuroQoL group during the period of the trial [49]. The summary of the EQ-5D utility scores at each follow-up point by each treatment arm will be presented and the overall difference in utilities between the arms will be examined through an appropriate model. QALYs will be estimated using the area-under-the-curve analysis [50].

Health care resource data will be collected at different time points using patient self-administered questionnaires and hospital CRFs and compared with the relevant Health Resource Group. Cost per patient will be estimated by multiplying the use of resource use by their associated unit costs. Unit costs will be sourced from the NHS Reference Costs databases [51], the Personal Social Services Research Unit [52], the *British National Formulary* [53] and other published literature. Though the primary perspective of the cost analysis will be that of the NHS and Personal Social Services, data on indirect costs associated with patient private expenses, days lost from work and from normal activities (e.g. household chores, shopping) will also be collected and included in a secondary analysis.

Cost and QALY data will be synthesised to generate an incremental cost-effectiveness ratio, which is defined as the ratio of the mean difference in costs to the mean difference in QALYs between treatments. Multivariate regression models will be used to assess the heterogeneity in costs, QALYs and cost-effectiveness. Multiple imputation techniques will be used to address the statistical issues related to the presence of missing data in the economic evaluation [54]. In order to characterise the uncertainty in the data, structural, scenario and probabilistic sensitivity analyses will be conducted. The uncertainty will be presented using a cost-effectiveness acceptability curve which shows the probability of the surgical interventions being more cost-effective than ESP conditional on a maximum value being attached to an additional unit of health outcome [55].

For the longer term, and if it is appropriate, QALYs for each patient will be calculated by extrapolating results of the trial-based analysis to a longer time horizon. For example, by assessing the long-term impact on health-related quality of life and costs at 5 years as at this time around 40% of patients continue to have from mild-to-severe symptoms [4]. The potential value of further research in this area will be considered [56].

### Nested qualitative study

The trial will be supplemented by a qualitative study that will focus on the following objectives to complement the trial objectives: to explore the experience and acceptability of the different treatments to patients and health care professionals; and to provide important patient-centred insight to further guide clinical decision-making. We will also explore, as a subsidiary aim, participants’ experiences of taking part in a surgical trial.

Up to 45 of the trial participants will take part in the interviews and will be drawn from those who have experienced the three trial treatments. As gender and diabetes can have an impact on outcome from shoulder surgery [8] effort will be made to include interviews with both men and women, and those with and without diabetes. The interviews will take place at approximately 12 months after enrolment into the trial to mirror the primary time point in the analysis. Interviews will be semi-structured with open questions. A flexible interview schedule will be developed following a literature review, discussions with the research team, patients with frozen shoulder, a physiotherapist and surgeon with expertise in this area. Interviews will be open and flexible to allow participants the opportunity to introduce new topics, and generate a detailed, personal perspective upon the topic [57]. We will continue to interview men and women to a point of theoretical saturation [58]; in short, until no further useful conceptual categories emerge.

Interviews will be ideally undertaken face-to-face although, given the geographic spread of participants, it may be more practical to perform some interviews by telephone or face-time interviews on-line (e.g. Skype); it is expected that up to 50% of interviews will be performed in this way. Interviews will be conducted by a qualitative researcher and audio-recorded with permission; and recordings will be transcribed in full.

We will also interview 10 to 15 health care professionals (physiotherapists and surgeons) about their experience of delivering the treatment. An interview schedule will be developed to address areas such as: clinical decision-making; treatment preference; and barriers to, and facilitators of, positive outcome of treatments.

To reflect the exploratory nature of this study, and to ensure that the participants’ perspective is at the heart of any insight generated, data analysis will be undertaken inductively [59]. However, our analytic approach is underpinned by constructivism, which takes the stance that qualitative research findings are not ‘discovered’ but co-constructed by the researcher and participant [58]. Qualitative finding are thus an interpretation. Through a process of constantly comparing data [58], we will develop initial tentative conceptual categories and then further abstract these categories into overarching themes that will help us to understand the experience. We will use NVivo qualitative data analysis software version 11 to assist our organisation of qualitative analysis. All interviews will be analysed by the researcher conducting the interviews with a second qualitative researcher coding a subset of interviews and commenting on the development of conceptual categories. The aim of this is not to reach consensus but to challenge the emerging interpretation and ensure interpretive rigour [60]. We will develop our conceptual categories and themes collaboratively in team meetings.

### Update of systematic review

To place the RCT findings in the context of current evidence at the end of the trial, we will update the HTA systematic review about the management of frozen shoulder incorporating the proposed RCT and any other new RCTs completed since the original searches were undertaken [5]. The review protocol will be registered on an international prospective register of systematic review (PROSPERO) prior to the analysis of the trial being undertaken. We will update the results of the review incorporating UK FROST and any other new RCTs. Any differences between the updated and original review will be highlighted.

### Monitoring

#### Data monitoring

A DMEC, independent of the funding body, sponsor and trial team, will be established and follow the research governance guidelines provided by the funding body and a charter. Only the DMEC will have access to the unblinded comparative data from the trial. The DMEC will monitor the data and make any recommendations about (dis)continuation of the trial to the independent TSC. The TSC will meet after the DMEC and provide overall supervision of the trial on behalf of the sponsor and funder.

There are no planned interim analyses for the trial or stopping guidelines. There will, however, be an internal pilot study from which the data will contribute to the final analyses. The primary reason for this pilot study is for the DMEC and TSC to check the assumptions about the feasibility of the trial to continue as planned or not, particularly concerning participant adherence to the ESP and intra-articular steroid injection.

#### Adverse event management

Adverse events are any untoward medical occurrence in a trial participant and may be a non-serious adverse event (AE) or a serious adverse event (SAE). At participating sites, all SAEs will be recorded and returned to the ‘UK FROST’ central office within 24 h of the investigator becoming aware of them. Once received, causality and expectedness will be determined by the CI. SAEs that are unexpected and related to the trial will be notified to Research Ethics Committee (REC) within 15 days for a non-life threatening event and within 7 days for a life-threatening event. For non-serious AEs, the central office will be notified within 5 days of the event being known. All (S)AEs will be reported to the DMEC, TSC and TMG. Expected adverse events for this shoulder condition include: infection; bleeding; delayed wound healing; conversion of planned day-case procedure to overnight stay for control of pain; post-procedural worsening of shoulder pain; injury to adjacent structures like nerve, tendon, bone or joint; recurrent stiffness requiring further treatment; and transient hyperglycaemia, steroid flare or joint sepsis following corticosteroid injection; injuries related to heating or cooling of tissues. Follow-up reports a month later will be reviewed by the CI to ensure that adequate action has been taken and progress made. We will only record (S)AEs that are related to the affected shoulder up to 12 months from randomisation.

#### Auditing

South Tees Hospitals NHS Foundation Trust will be the sponsor for this project. This study will be fully compliant with the Research Governance Framework and Good Research Practice [45]. The TMG will review core trial processes/progress on a quarterly basis which includes representation from the sponsor. No site visits to monitor progress are planned but could be initiated depending on progress made as reviewed at the TMG.

### Ethics and dissemination

#### Research Ethics Committee approval

REC approval was granted on 18 November 2014 (NRES Committee North East – Newcastle and North Tyneside 2). Health Research Authority (HRA) approval for the study with an existing UK wide review was granted on 15 June 2016.

#### Protocol amendments

Any substantial amendments will be submitted to the REC having been agreed with: the funding body, sponsor, TSC, DMEC, TMG and the Research Governance Committee for the Department of Health Sciences, University of York. Minor modifications to the protocol will be agreed with the TMG and sponsor before submission for approval to REC. All amendments will be implemented in the NHS organisations in agreement with the guidance and approval of the HRA. All amendments will be listed in the published final report to the funding body.

#### Consent or assent

Written consent will be obtained for all trial participants by the trained local RN or clinician using a detailed patient information sheet developed with the help of service users and explaining the risks and benefits clearly. For the nested qualitative study we will write to the trial participants and health care professionals about taking part in the interviews and to confirm this with written consent. Information sheets will again be provided.

#### Confidentiality

All data will be identified by a coded ID (identification) number to maintain participant confidentiality. All study-related paper forms will be stored securely in the University of York and, after a period of time, transferred to a secure, off-site facility below ground with no moisture, no vermin and virtually no fire risk. Access to the archive area is via a security controlled mine shaft with no outward markings to advertise its presence. All electronic records will be stored on a password-protected server and will be anonymous of identifiable information. All participant information will be stored in locked cabinets in areas with restricted access (i.e. alarmed area) at the University of York. For the qualitative interviews, recordings and transcripts will be kept anonymised and kept in a locked office at the University of Oxford. Any quotation that could clearly be used to identify a person will not be used in the dissemination of findings. Participants’ data may be reviewed by authorised persons on the research team or other authorised people to verify that the study is being carried out correctly all of whom will have a duty of confidentiality. Trial participants will consent on enrolment to permit this authorised review. All names and other identifying information will be removed before the data is analysed and the results presented to the medical community at conferences and in scientific journals.

#### Declaration of interests

The independent members of the DMEC and TSC will sign a form to confirm that they have no competing interests to declare. Competing interests of the authors are presented at the end of the protocol.

#### Access to data

The Secretary of State for Health (the Authority) has the right to access data during the research and will respect existing guidance on confidentiality for any data which it obtains. Data that will be shared internally within the trial team will be blinded of any identifying participant information to ensure confidentiality. When there is a request to use UK FROST data from external researchers this will be notified to the TMG. When the TMG agrees to an external request for data the approval for this will be confirmed with the sponsor and the funding body. Data will be provided in an anonymised format and securely transferred to the requester.

#### Ancillary and post-trial care

The trial treatments are all routinely available in the NHS. Therefore, any ancillary care of post-trial care that includes continuing treatment of a frozen shoulder should be accessible to all trial participants in discussion with their clinician. If a trial participant wishes to complain formally, they will be advised to do this through the usual NHS Complaints Procedure. If a patient is harmed and this is due to someone’s negligence then they may have grounds for legal action or compensation against the sponsor (in respect of harm arising out of participation in the trial) or the NHS (in respect of any harm which has resulted from the treatment received).

#### Dissemination policy

The trial results will be disseminated regardless of the magnitude or direction of effect [61] to key stakeholders and patients in several ways: peer-reviewed journal including the HTA monograph; to Commissioning Reference Groups; presented at key scientific meetings; made available on specialist websites; feedback to trial participants, update the entry on Wikipedia and write the Map of Medicine entry on frozen shoulder management; through press releases at collaborating universities; and we will explore non-academic routes to dissemination such as patient.co.uk.

The criteria for authorship will be taken from the International Committee of Medical Journal Editors [62]. Those who do not meet the authorship criteria but contributed to aspects of the study design or drafting of work will be acknowledged as contributors. Those who were solely involved in trial conduct (e.g. staff at recruiting sites) will be acknowledged as collaborators. When a journal permits we will list all authors rather than use a group name.

This protocol is being made publicly available. The full trial report will be submitted to the funding body and for publication in a peer-reviewed journal. The full trial report will be open access and made available as a permanent archive in the NIHR Journals Library. At the time of publishing the protocol there was no plan to make the anonymised participant-level dataset and statistical code for generating the results publicly available. After publication of the main trial findings, however, an external request that is made for this data and code will be agreed by the TMG and confirmed with the sponsor and funding body.

## Discussion

This research will further knowledge and understanding of the impact of frozen shoulder on the clinical and cost-effectiveness of physiotherapy and the more invasive surgical treatments commonly used. There is increasing awareness that the findings from RCTs are more valuable when considered in the context of existing evidence on all treatments of interest. Therefore, we will update the recent HTA systematic review about the management of the frozen shoulder [5] but with a focus on RCTs of the interventions included in the trial. Contextualising our research findings in this way will help inform clinical practice in the NHS and provide direction for future research in this area.

## Trial status

The current REC approved version of the protocol is version 4.0 (15 November 2016). This manuscript is a re-structured and edited version of the current REC-approved protocol to comply with the SPIRIT guidelines. The first patient was recruited into the trial on 14 April 2015 and recruitment should be complete for the end of December 2017.

## Additional file

Additional file 1: The Standard Protocol Items Recommendations for Interventional Trials (SPIRIT) Statement 2013 have been followed for the completion of the protocol (see also Additional file 1: SPIRIT 2013 Checklist: recommended items to address in a clinical trial protocol and related documents). (DOC 120 kb)

## Abbreviations

ACR: Arthroscopic capsular release
AE: Adverse event
BESS: British Elbow and Shoulder Society
CI: Chief investigator
CONSORT: Consolidated Standard of Reporting Trials
CRF: Case Report Forms
DMEC: Data Monitoring Ethics Committee
EQ-5D: EuroQol 5 dimensions
ESP: Early Structured Physiotherapy
HRA: Health Research Authority
HTA: Health Technology Assessment
ITT: Intention-to-treat
MUA: Manipulation under anaesthesia
NHS: National Health Service
NIHR: National Institute for Health Research
OSS: Oxford Shoulder Score
PI: Principal investigator
PPP: Post-procedural physiotherapy
QALY: Quality-adjusted-life-year
*Quick*DASH: *Quick* Disabilities of Arm Shoulder and Hand
RCT: Randomised controlled trial
REC: Research Ethics Committee
RN: Research nurse
SAE: Serious adverse event
SIV: Site Initiation Visits
TMG: Trial Management Group
TSC: Trial Steering Committee
UK: United Kingdom
UK FROST: United Kingdom Frozen Shoulder Trial
YTU: York Trials Unit
